# Different intensities of aerobic training for patients with type 2 diabetes mellitus and knee osteoarthritis: a randomized controlled trial

**DOI:** 10.3389/fendo.2024.1463587

**Published:** 2024-09-02

**Authors:** Chi Su, Lihua Huang, Shaochen Tu, Shengdi Lu

**Affiliations:** ^1^ Department of Orthopedics, Deyang Hospital Affiliated to Chengdu University of Traditional Chinese Medicine, Deyang, Sichuan, China; ^2^ Department of Rehabilitation, Shanghai Sixth People’s Hospital Affiliated to Shanghai Jiao Tong University School of Medicine, Shanghai, China; ^3^ Department of Orthopedics, Fuzhou Second General Hospital, Fujian, China; ^4^ Department of Orthopedics, Shanghai Sixth People’s Hospital Affiliated to Shanghai Jiao Tong University School of Medicine, Shanghai, China

**Keywords:** aerobic training, type 2 diabetes mellitus, knee osteoarthritis, KOOS, HbA1c

## Abstract

**Objective:**

The purpose of this study was to compare different intensities of aerobic exercise for patients with knee osteoarthritis (KOA) and type 2 diabetes mellitus (T2DM) in terms of glycemic control, pain relief, and functional outcomes.

**Methods:**

A prospective randomized open-label parallel multicenter clinical trial conducted at two hospitals in Shanghai and Sichuan that included 228 patients with type 2 diabetes mellitus (T2DM) and knee osteoarthritis (KOA). Enrollment occurred between January 2021 and February 2023, and follow-up was completed in September 2023. Participants were randomized to threshold training/high-intensive stationary cycling training (n=76), intensive endurance/moderate-intensive stationary cycling training (n=77), and regular rehabilitation programs (n=75). The primary outcome at the 6-month follow-up was the HbA1c level. Key secondary outcomes included the Knee Injury and Osteoarthritis Outcome Score (KOOS) subscale of pain and quality of life.

**Results:**

Of 228 patients, 212 (93%) completed the trial. The mean adjusted (sex, baseline BMI, and baseline outcome measures) HbA1c level at the 6-month follow-up decreased significantly in the high-intensive training group compared with other groups (high-intensity group vs. control group; difference, 0.51%, 95% confidence interval, 0.05% to 1.15%). Mean KOOS subscales of pain and quality of life were statistically significantly different between the control group and moderate-intensity or high-intensity groups, but no statistical differences were noted between the different intensities of aerobic exercise. Patients in all groups achieved a greater reduction in BMI but no significant differences were observed between groups.

**Conclusion:**

In KOA and T2DM patients, high-intensity stationary cycling can significantly improve glycemic control compared with moderate-intensity and regular rehabilitation programs. However, high-intensity stationary cycling does not exert a superior effect on pain relief and functional improvement for KOA compared with moderate-intensity and regular rehabilitation programs.

## Introduction

Multimorbidity is a rising public health challenge with important implications for health management and policy. The most common multimorbidity pattern is the combination of cardiometabolic and osteoarticular diseases, exemplified by the highly prevalent co-occurrence of type 2 diabetes mellitus (T2DM) and osteoarthritis (1). The relationship between T2DM and knee osteoarthritis (KOA) has garnered attention due to the overlapping prevalence and shared risk factors, such as obesity and advanced aging. Research indicates a significant association between T2DM and KOA. A study that included populations with type 1 DM (T1DM) and T2DM observed a notably higher association of KOA with T2DM, with an odds ratio (OR) suggesting a more than double likelihood of individuals with T2DM developing KOA compared with those without T2DM. Interestingly, this association was stronger among non-obese individuals, highlighting the potential impact of diabetes beyond the effects of obesity alone (2). There were other studies that collectively underscored the significant correlation between T2DM and KOA, suggesting that the mechanisms linking these conditions go beyond simple risk factors like obesity (3, 4).

Exercise is considered a cornerstone of treatment for T2DM alongside diet and medication of proven efficacy (5, 6). Although the effectiveness of exercise in improving glycemic control, blood lipid profiles, and other outcomes in this group is well documented (7–9), there is less certainty about the relative effects of different types of exercise. Aerobic exercise is traditionally the most studied exercise (8) and recruits large groups of muscles and includes brisk walking, cycling, swimming, and jogging.

For KOA, the most common form of aerobic land‐based exercise found in the literature was stationary bicycle (10), as it is a low weight bearing and non-impact form of physical activity. It has been shown that stationary cycling performed over 10 to 12 weeks leads to a reduction in knee pain and stiffness, and an improvement in walking speed and distance in individuals with KOA (11, 12). Positive benefits of rehabilitation caused by cycling could be attributed to improvements in leg muscular power output and the dynamic range of motion (13). For individuals with KOA, both low- and high-intensity cycling are reported to be therapeutically beneficial (11, 12).

Patients with both KOA and T2DM present unique pathophysiological challenges. KOA leads to joint pain and reduced mobility, making exercise more difficult, and T2DM requires effective glycemic control, which is often achieved through physical activity. Understanding the appropriate exercise intensity can help tailor interventions that effectively manage both conditions without exacerbating either.

Exercise is a cornerstone in the management of both KOA and T2DM (14–17). However, the optimal intensity of aerobic exercise that maximizes benefits for both conditions simultaneously is not well-established. Research in this area could identify exercise protocols that improve joint function, reduce pain, and enhance insulin sensitivity, leading to better overall health outcomes (14–17). In addition, incorrect exercise intensity may lead to increased joint pain or injury in KOA patients or inadequate glycemic control in T2DM patients. Establishing evidence-based guidelines for aerobic exercise intensity can prevent these adverse effects, ensuring that exercise regimens are safe and effective.

To our knowledge, no studies have examined the intensity of aerobic exercise in patients with multimorbidity of both KOA and T2DM. The purpose of this study was to compare different intensities of aerobic exercise for patients with KOA and T2DM in terms of glycemic control, pain relief, and functional outcomes.

## Methods

### Study design

This is a prospective randomized open-label parallel multicenter clinical trial. Eligible participants were recruited from the Shanghai Sixth People’s Hospital Affiliated to the Shanghai Jiao Tong University School of Medicine, Deyang Hospital Affiliated to the Chengdu University of Traditional Chinese Medicine, and Fuzhou Second General Hospital and were divided into the intervention group and the control group. The allocation ratio was 1:1:1 (two intervention groups and one control group). This trial was registered at chictr.org.cn before participants were recruited (ChiCTR2100042872) and was approved by the Institutional Review Board of the Shanghai Sixth People’s Hospital Affiliated to Shanghai Jiao Tong University School of Medicine [IRB No: 2019-KY-063(K)]. Other trial sites acknowledged the approval. All participants provided written informed consents.

### Study participants

KOA was confirmed according to the criteria from the National Institute for Health and Clinical Excellence (18): patients can be diagnosed with KOA if they are 45 years or older, have movement-related joint pain, and either no morning knee stiffness or stiffness of 30 min or less.

The definition of type 2 diabetes in the present study was formulated according to the SUPREME-DM (19) criteria as follows: a) one or more of the International Classification of Disease, Ninth Revision, Clinical Modification (ICD-9-CM) codes and Tenth Revision, Clinical Modification (ICD-10-CM) codes for type 2 diabetes associated with in-patient encounters; b) two or more ICD codes associated with outpatient encounters on different days within 2 years; c) a combination of two or more of the following associated with outpatient encounters on different days within 2 years: 1) ICD codes associated with outpatient encounters; 2) a fasting glucose level ≥126 mg/dl; 3) a 2-h glucose level ≥200 mg/dl; 4) random glucose ≥200 mg/dl; 5) HbA1c ≥6.5%; and 6) a prescription for an antidiabetic medication.

### Interventions

The participants in the intervention group were instructed to undertake regular rehabilitation plus stationary cycling exercises every day for at least 30 min at different targeted heart rates. All participants in this study were asked to complete a graded exercise test (GXT) (19, 20) before the intervention. Submaximal anchor measurements were derived from the GXT result to prescribe exercise intensity. The submaximal anchor measurement model we used in this study included five exercise intensity levels (L1–L5) (20, 21) (**Table 1**). In this study, intensity levels 2 (extensive endurance/moderate-intensive group) and 3 (intensive endurance/high-intensive group) were used for our evaluation for patients with KOA and T2DM in terms of glycemic control, pain relief, and functional outcomes. For the control group, the participants were only instructed on regular rehabilitation. A professional healthcare group provided a comprehensive rehabilitation program to the participants in the intervention and control groups (**Supplementary Figure 1**). The rehabilitation program was delivered through a smartphone app (22–24) (device: Joymotion software, Shanghai Medmotion Medical Management Co., Ltd., Shanghai, China.), which provided participants with exercise instructions, feedback on their training performance, and real-time two-way video and audio interaction with the physical therapists (PTs). The app was installed by a technician on the day of the first visit. PTs at the rehabilitation center initiated the conference at the appointed time scheduled with the participants every week. The app provides daily rehabilitation exercises with detailed instructions and records the exercise completion rates. The rehabilitation program was prescribed by the supervising PTs and assigned to the participants as “daily tasks”. The content of the rehabilitation program is illustrated in the **Supplementary Table 1**.

**Table 1:** The five aerobic training levels based on the first and second lactate threshold (LT1/LT2) derived from a graded exercise test.

Aerobic training zone	Level 1 - recovery	Level 2 - extensive endurance	Level 3 - intensive endurance	Level 4 - threshold training	Level 5 - interval training
Heart rate (% of HRmax)	65–75%	75–80%	80–85%	85–92%	> 92%
Blood lactate (mmol.L^-1^)	< 2.0	2.0–2.5	2.5–3.5	3.5–5.0	> 5.0
Rating of perceived exertion (RPE) (6–20)	< 11	11–12	13–14	15–16	17–19
Relative to sub-maximal anchor	< LT1	LT1 < LT2	LT1 < LT2	> LT2	> LT2

Each level is characterized by a percentage of the maximum heart rate (% of HRmax), an absolute blood lactate value, a rating of perceived exertion, and the relationship with a submaximal anchor^15,16^.

### Outcomes

The primary outcome was the pre-post changes of HbA1c. The major secondary outcome was changes in the Knee Injury and Osteoarthritis Outcome Score (KOOS) from baseline. The KOOS is a patient-reported outcome measurement system used to evaluate short-term and long-term symptoms and function in individuals with knee injuries and osteoarthritis. The score consisted of five separately scored subscales: pain, symptoms, function in daily living (ADL), function in sport and recreation (Sport/Rec), and knee-related quality of life (QoL). The score ranges from 0 to 100 with 0 representing extreme problems and 100 representing no problems. Other secondary outcomes included changes in the body mass index (BMI).

### Sample size

We aimed to detect a difference of 0.5% in HbA1c change from baseline to achieve an 80% power at a significance level of 0.05, and considering a 10% dropout rate, approximately 70 participants were required per arm.

### Randomization, treatment, and follow-up

The participants were randomly assigned to the intervention and control groups in a 1:1:1 ratio. The randomization sequence was generated by the institutional staff and was concealed from the PTs during follow‐up. Anthropometric and clinical data were collected from the participants at baseline and at the third and sixth months after the intervention; these data included sex, marital status, family income, use of a walking aid, BMI, and history of diseases and medications. Height and weight were measured using standardized methods. BMI was calculated as the weight in kilograms divided by the squared height in meters. Other data were collected from questionnaires.

The follow-up period lasted for 6 months. During the follow-up period, participants in the intervention group were instructed by Joymotion on how to carry out the rehabilitation program plus stationary cycling for at least 30 min every day. The PTs were directed to evaluate the methods and provide additional help to the participants to correct and improve their rehabilitation program based on the online platform provided by Joymotion. The timing of stationary cycling was also monitored and the records were requested to be uploaded. The participants in the control group only used Joymotion for the rehabilitation program without further instruction and demands with regard to the cycling training. The rehabilitation goal for all the participants in this trial was prespecified as a significant improvement in symptoms.

### Blinding

No blinding was performed in this trial. Only the analyst who assessed the outcomes was blind to this trial.

### Statistical analysis

The analysis was conducted according to the intention-to-treat principle, with multiple imputed data for participants with missing data under the assumption that data were randomly missing. Continuous outcomes were reported as the least squares means and standard errors. Mixed linear models for repeated measures adjusted for sex, age, and KL grade were employed to analyze the change from baseline, including participants as random effects, with fixed effect factors for the group and week, and the corresponding interaction. Sensitivity analyses were performed for the primary and key secondary outcomes at month 6 by repeating the primary analyses on the per-protocol population predefined as participants with satisfactory adherence and without major protocol deviations. The group differences between least squares means were reported with two-sided 95% confidence intervals (CIs), and a two‐tailed *P* < 0.05 was defined as significant. False discovery rate correction was conducted for multiple testing. Counting data were reported as a percentage. Statistical significance was analyzed using a chi-square test, and *P* < 0.05 was defined as significant. All data were analyzed using R version 4.2.2 (R Foundation for Statistical Computing, Vienna, Austria).

## Results

### Baseline characteristics of the study participants

**Figure 1:**
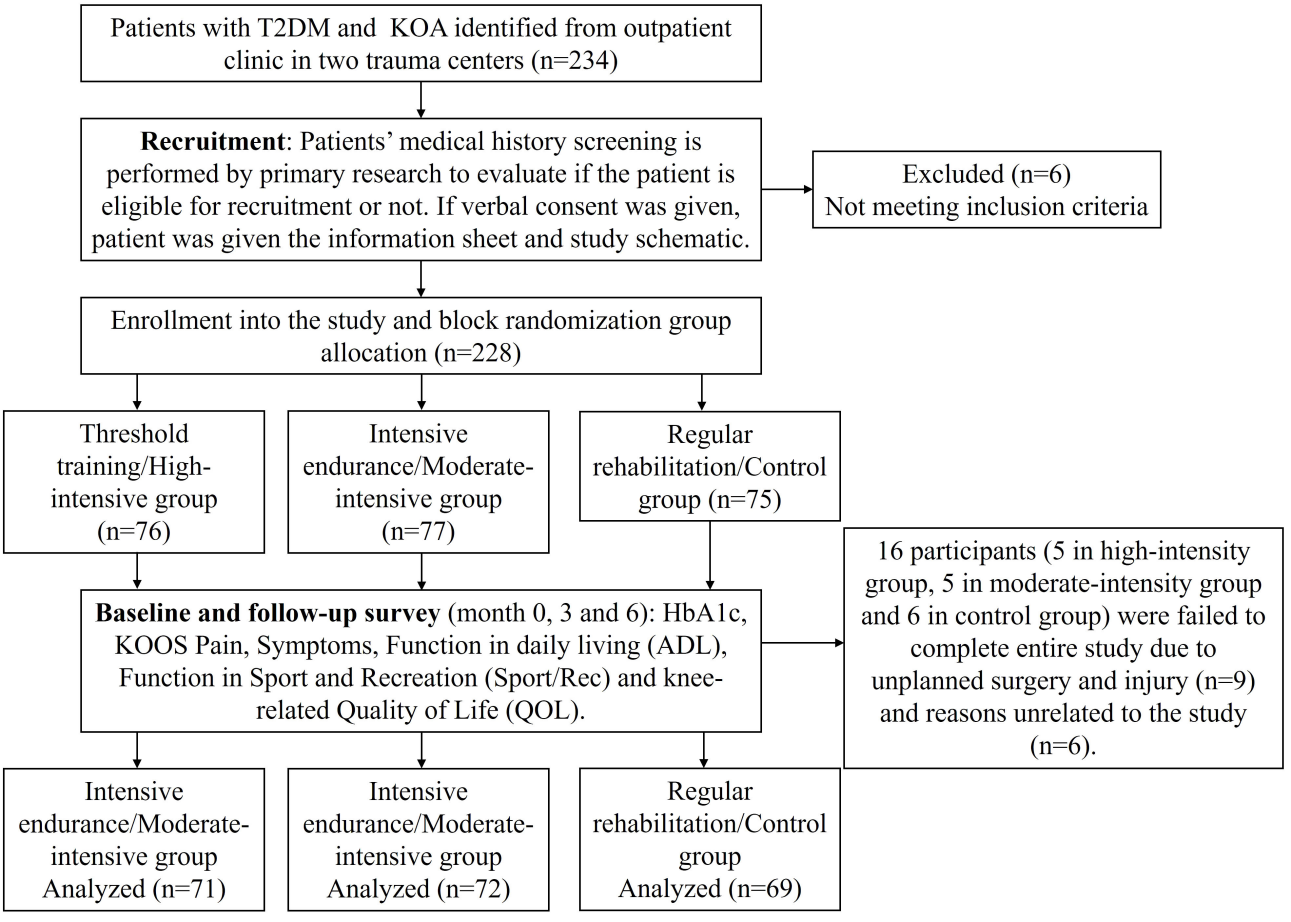
Flowchart of the study.

Of the 234 participants who underwent screening in this study, 228 were enrolled into the final analysis and randomized. Of these, 77 were in the intensive endurance/moderate-intensive group, 76 were in the threshold training/high-intensive group, and 75 were in the control group. Of these, 212 (93%) completed the full follow-up visits. Sixteen participants (five in the high-intensity group, five in the moderate-intensity group, and six in the control group) failed to complete the entire study due to unplanned surgery and injury (n=9) and reasons unrelated to the study (n=6) (**Figure 1**).

The baseline characteristics are shown in **Table 2**. The mean age was 61.5 ± 5.8 years. The mean BMI at baseline was 30.4 ± 4.5 kg/m^2^.

**Table 2:** Demographic and clinical characteristics of the study participants at baseline.

Characteristics	High-intensity group (N=76)	Moderate-intensity group (N=77)	Control group (N=75)
Age (years), mean (SD)	61.4 (5.2)	61.7 (6.2)	61.2 (6.0)
Gender, male (%)	36 (48.0)	37 (48.7)	39 (50.6)
BMI (kg/m^2^), mean (SD)	30.8 (4.3)	30.3 (5.0)	30.1 (4.2)
Obesity (BMI ≥30, %)	43 (57.3)	39 (51.3)	39 (50.6)
Education level			
≥ High school (%)	41 (54.7)	46 (60.5)	40 (51.9)
Insurance type (%)			
Government	60 (80.0)	58 (76.3)	61 (79.2)
Commercial	4 (5.3)	5 (6.6)	4 (5.2)
Self-financial	12 (14.7)	13 (17.1)	12 (15.6)
HbA1c level (%)	9.0 (1.1)	8.8 (1.3)	8.6 (1.2)
Comorbid illness* (%)			
Hypertension	52 (69.3)	47 (61.8)	51 (66.2)
Cardiovascular disease	14 (18.7)	16 (21.1)	13 (16.9)
Arthritis in other joints	16 (21.3)	18 (23.7)	17 (22.1)
Kellgren–Lawrence grade**			
2	35 (46.7)	36 (47.4)	37 (48.1)
3	32 (42.7)	27 (35.5)	29 (37.7)
4	8 (10.7)	13 (17.1)	11 (14.3)
Patellofemoral OA, severity 1,2 (mild-moderate)***	61 (81.3)	60 (78.9)	58 (75.3)
Paracetamol and NSAID (%)	53 (70.7)	57 (75.0)	56 (72.7)
Walking aid used	1 (1)	0	1 (1)

*Reported on a self-administered health history questionnaire (with the exception of patellofemoral OA) as conditions diagnosed by a health care professional. With comorbid illnesses that could exclude patients from participation, final approval or denial for participation provided after patient evaluation by a study physician.

**The Kellgren–Lawrence scale ranges from 0 to 4. A grade of 2 or greater indicates definite osteoarthritis on a posteroanterior weight-bearing radiograph. A grade of 2 indicates definite osteophytes and possible joint space narrowing; grade 3, multiple osteophytes, definite joint space narrowing, sclerosis, and possible bony deformity; and grade 4, large osteophytes, marked definite joint space narrowing, severe sclerosis, and definite bony deformity.

*** Patellofemoral OA measured from a skyline view radiograph using the OARSI scale (0, none; 1, mild; 2, moderate; 3, severe). Patients with severe (JSW, 3) patellofemoral OA were excluded. One patient was missing baseline skyline view radiographs.

### Changes in HbA1c

Pre-post changes in HbA1c were significant at the 6-month follow-up in each group. Participants in the threshold training group achieved a significant decrease in HbA1c compared with participants in the intensive endurance group and control group (**Table 3**), exceeding the minimal clinically important difference of 0.5% (25).

**Table 3:** Primary, key secondary, and other outcomes at 6 months in the intention-to-treat population.

Outcome	High-intensity group (N=76)	Moderate-intensity group (N=77)	Control group (N=75)	Mean difference (95% CI)	*P* value
Primary outcomes					
HbA1c level					
6-month adjusted means from baseline (95% CI)*	1.13 (0.93, 1.33)	0.72 (0.50, 0.94)	0.61 (0.40, 0.83)		
High-intensity vs. control				0.51 (0.05, 1.15)	.01
High-intensity vs. moderate-intensity				0.38 (0.05, 0.74)	.04
Moderate-intensity vs. control				0.10 (-0.22, 0.43)	.10
Key secondary outcomes					
KOOS pain					
6-month adjusted means from baseline (95% CI)	3.3 (2.8, 3.8)	2.9 (2.0, 3.8)	1.6 (0.9, 2.3)		
High-intensity vs. control				1.7 (0.4 to 2.8)	.02
High-intensity vs. moderate-intensity				0.6 (-0.8 to 1.8)	.45
Moderate-intensity vs. control				1.3 (0.3 to 2.3)	.02
KOOS QoL					
6-month adjusted means from baseline (95% CI)	6.0 (3.9, 8.0)	4.7 (3.4, 5.0)	2.5 (1.7, 3.3)		
High-intensity vs. control				3.5 (1.0 to 6.0)	.01
High-intensity vs. moderate-intensity				1.2 (-0.7 to 3.0)	.48
Moderate-intensity vs. control				2.3 (0.1 to 4.5)	.04
Other secondary outcomes					
KOOS symptoms					
6-month adjusted means from baseline (95% CI)	7.9 (6.3, 9.5)	6.5 (4.7, 8.3)	5.1 (3.6, 6.6)		
High-intensity vs. control				2.8 (0.1 to 5.9)	.04
High-intensity vs. moderate-intensity				1.4 (-1.3 to 4.3)	.65
Moderate-intensity vs. control				1.4 (-1.7 to 4.5)	.79
KOOS ADL					
6-month adjusted means from baseline (95% CI)	13.6 (10.8, 16.4)	10.9 (8.3, 13.5)	8.7 (6.9, 10.5)		
High-intensity vs. control				4.9 (1.1 to 8.7)	.02
High-intensity vs. moderate-intensity				2.7 (-2.5 to 7.9)	.61
Moderate-intensity vs. control				2.2 (-2.0 to 6.4)	.85
KOOS sport/Rec					
6-month adjusted means from baseline (95% CI)	5.1 (3.7, 6.5)	3.6 (2.4 to 4.8)	2.2 (1.5 to 2.9)		
High-intensity vs. control				2.9 (0.8 to 5.0)	.02
High-intensity vs. moderate-intensity				1.5 (-1.0 to 4.0)	.79
Moderate-intensity vs. control				1.4 (-0.4 to 3.2)	.65
BMI					
6-month adjusted means from baseline (95% CI)	2.8 (1.9 to 3.7)	2.3 (1.5 to 3.1)	1.8 (1.1 to 2.5)		
High-intensity vs. control				1.0 (-0.4 to 2.4)	.63
High-intensity vs. moderate-intensity				0.5 (-0.9 to 1.9)	.70
Moderate-intensity vs. control				0.5 (-0.8 to 1.8)	.88

KOOS, Knee injury and Osteoarthritis Outcome Score; QoL, quality of life; Rec, recreation; ADL, ability of daily life.

Analyses were conducted to multiply imputed data for participants with missing data under the assumption that data were randomly missing.

### Changes in the KOOS

A multivariate generalized linear model defined KOOS improvement as a dependent variable and the control group as the reference. After adjustments in age, gender, and KL grade, the results showed that the patients with high intensity were statistically significantly different between the control group and moderate-intensity group with regard to the pain subscale of the KOOS. In addition, patients in the moderate-intensity group showed statistically superior results to patients in the control group (**Table 3**).

**Figure 2:**
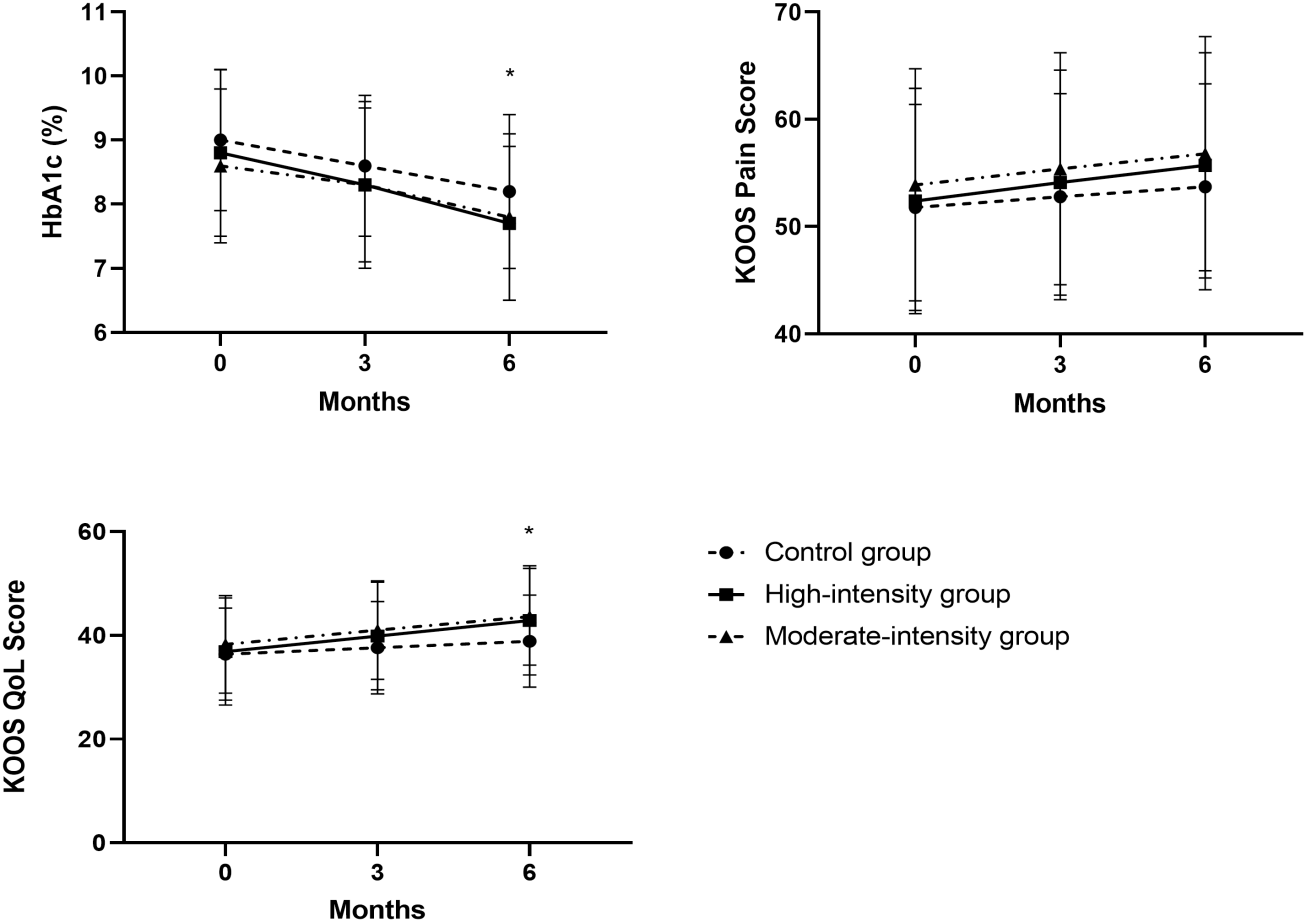
Trajectories of HbA1c and the KOOS subscales of pain and QoL in the PP population. For the KOOS subscales, high values represent better outcomes. Data points represent means at each follow-up time point (error bars indicate standard deviation). *P < 0.05. KOOS, Knee Injury and Osteoarthritis Outcome Score; PP, per protocol; QoL, quality of life.

The average changes in the KOOS QoL score from baseline to 6 months were 6.0 (95% CI, 3.9 to 8.0) in the high-intensity group, 4.7 (95% CI, 3.4 to 5.0) in the moderate-intensity group, and 2.5 (95% CI, 1.7 to 3.3) in the control group (**Figure 2**). The high-intensity and moderate-intensity groups exhibited significant improvements compared with the control group, as shown in **Table 3**. However, no significant inter-group difference was noted. Regarding other KOOS subscales (KOOS symptoms, KOOS ADL, and KOOS sport/Rec), patients undergoing high-intensity training (HIT) demonstrated significantly better outcomes than those in the control group. However, no significant differences were observed between the high-intensity and moderate-intensity groups (**Table 3**). Participants undertaking moderate-intensity stationary cycling exhibited similar results to those on the regular rehabilitation program with regard to KOOS symptoms, KOOS ADL, and KOOS sport/Rec.

### Changes in BMI

Health outcomes are also presented in **Table 3**. Patients in all groups achieved a greater reduction in BMI but no significant differences were observed between groups.

### Adverse events and other outcomes

During the follow-up, no adverse events were reported.

## Discussion

Our randomized controlled trial involving T2DM patients indicated that stationary cycling improved the physical function of KOA, by improving muscle strength, compared with a regular rehabilitation program. Although participants in high-intensity stationary cycling had better functional results than participants in the control group, participants in different intensities of stationary cycling had similar results with regard to pain relief and functional improvement. We also found that high-intensity stationary cycling was more effective in glycemic control than moderate-intensity stationary cycling.

Osteoarthritis and T2DM are complex diseases influenced by genetic, demographic, and lifestyle factors, such as older age and obesity (26). Lifestyle intervention is considered a cornerstone in the treatment of osteoarthritis and T2DM (4–8, 10, 11). Among different types of lifestyle interventions, stationary cycling is primarily considered an aerobic activity. It involves continuous rhythmic movements of large muscle groups and is designed to improve cardiovascular fitness by increasing the heart rate and oxygen consumption over a sustained period. Additionally, it can help with muscle endurance in the lower body and does not add an extra burden on the knee joint. However, the standard for determining effective activity intensity remains controversial for patients with T2DM and KOA. In most studies of healthy individuals and diabetic populations, it is evident that a positive dose-response relationship exists between a higher exercise dose and improved physiologic changes, physical capacity, and performance (27, 28). In contrast, a different mechanism seems to be involved in patients with musculoskeletal pain, with previous studies showing inconsistent intensity-response relationships (29, 30).

Submaximal and maximal anchors have also been used in different models to define different training intensities (20, 31). The model we used in this study has five exercise intensity levels derived from GXT (19, 20). These levels can be further characterized by percentages of HRmax, blood lactate values, and ratings of perceived exertion (RPE) (**Table 1**). Participants were supposed to reach an 80–85% of HRmax and 85–92% of HRmax in two intervention groups, which resulted in a different effect of glycemic control in our study. In line with other studies, patients with a higher percentage of HRmax when participating in aerobic exercise had a better effect in glycemic control. In a previous systematic review by Umpierre et al., aerobic and resistance exercises decreased HbA1c by 0.73 and 0.57%, respectively (32). However, different intensities of aerobic exercise did not lead to different effects on pain relief or functional improvement in KOA in our study. There were some statistical differences favoring the high-intensity group in the domain of knee function at the end of the treatment and 3 months after intervention; however, none of these differences persisted at the 6-month follow-up. Notably, most variables numerically favored the high-intensity group, albeit not in a statistically or clinically meaningful way. Other reports have found similar results to our study with regard to the intensity-response relationships of aerobic training and KOA (33, 34).

Studies have shown that HIT increases insulin sensitivity and glycemic control through various mechanisms. For instance, HIT has been reported to improve muscle metabolic adaptations, regulate inflammation, and ameliorate lipid metabolism, all contributing to improved glucose homeostasis and insulin sensitivity (35–39). Additionally, although our study primarily focused on the short-term effects (6 months), it is essential to consider the potential long-term impact and safety of HIT, especially on joint health in KOA patients. Long-term HIT may lead to sustained improvements in insulin sensitivity but it is crucial to monitor for potential joint wear and injury risks. Thus, future studies should focus on the long-term adherence and safety of HIT programs in KOA patients to ensure they can benefit from the training without adverse effects.

Englund suggested that the modest benefits of exercise interventions for KOA could largely be attributed to the placebo effect, the disease’s natural progression, and statistical regression to the mean (40). The considerable sample size and extended duration of the study likely heightened the placebo response, particularly regarding the subjective experience of pain (41). Additionally, the significant pain reduction observed in the control group might explain why there was no substantial difference between the high-intensity strength training group and the control group (41). Some authors considered the use of exercise treatment in chronic pain conditions should be viewed as a form cognitive therapy, in which the goal is to modulate the feeling of pain and thus patients’ thoughts and feelings about it rather than increasing muscle strength and endurance (33, 41, 42). In a previous trial, placebo treatment matched the efficacy of exercise therapy^31^. Our study found no significant differences among high-intensity, moderate-intensity, and control groups over a 6-month intervention, possibly due to placebo effects increased by close supervision via an online platform. The attentive interaction between patients and the online platform may have overshadowed the expected dose-response benefits of exercise, which only became apparent when this direct attention ceased.

This study has several limitations. First, the results may be more generalizable to individuals who are comparable with the study sample, the majority of whom were men, obese, and had more than a high school education. Second, submaximal anchors have been used to define the domains of exercise, even though the majority of these methods have not been confirmed to elicit domain-specific physiological responses. Third, a trial with a larger sample size and long-term follow-up period is required to evaluate the effectiveness of exercise therapy in KOA and T2DM patients.

## Conclusion

Among KOA and T2DM patients, high-intensity stationary cycling has a significantly greater glycemic control capability than moderate-intensity stationary cycling and a regular rehabilitation program. However, high-intensity stationary cycling does not have a superior effect on pain relief and functional improvement in KOA compared with moderate-intensity stationary cycling and a regular rehabilitation program.

## Data Availability

The raw data supporting the conclusions of this article will be made available by the authors, without undue reservation.

## Supplementary material

Supplementary Figure 1 Rehabilitation program.

Supplementary Table 1 Primary and key secondary outcomes at 6 months in the per-protocol population.
