# Supplementary figures and images for: Different intensities of aerobic training for patients with type 2 diabetes mellitus and knee osteoarthritis: a randomized controlled trial
Source: Front Endocrinol (Lausanne). 2024 Sep 2;15:1463587. doi: 10.3389/fendo.2024.1463587 (PMC11402742; doi:10.3389/fendo.2024.1463587)

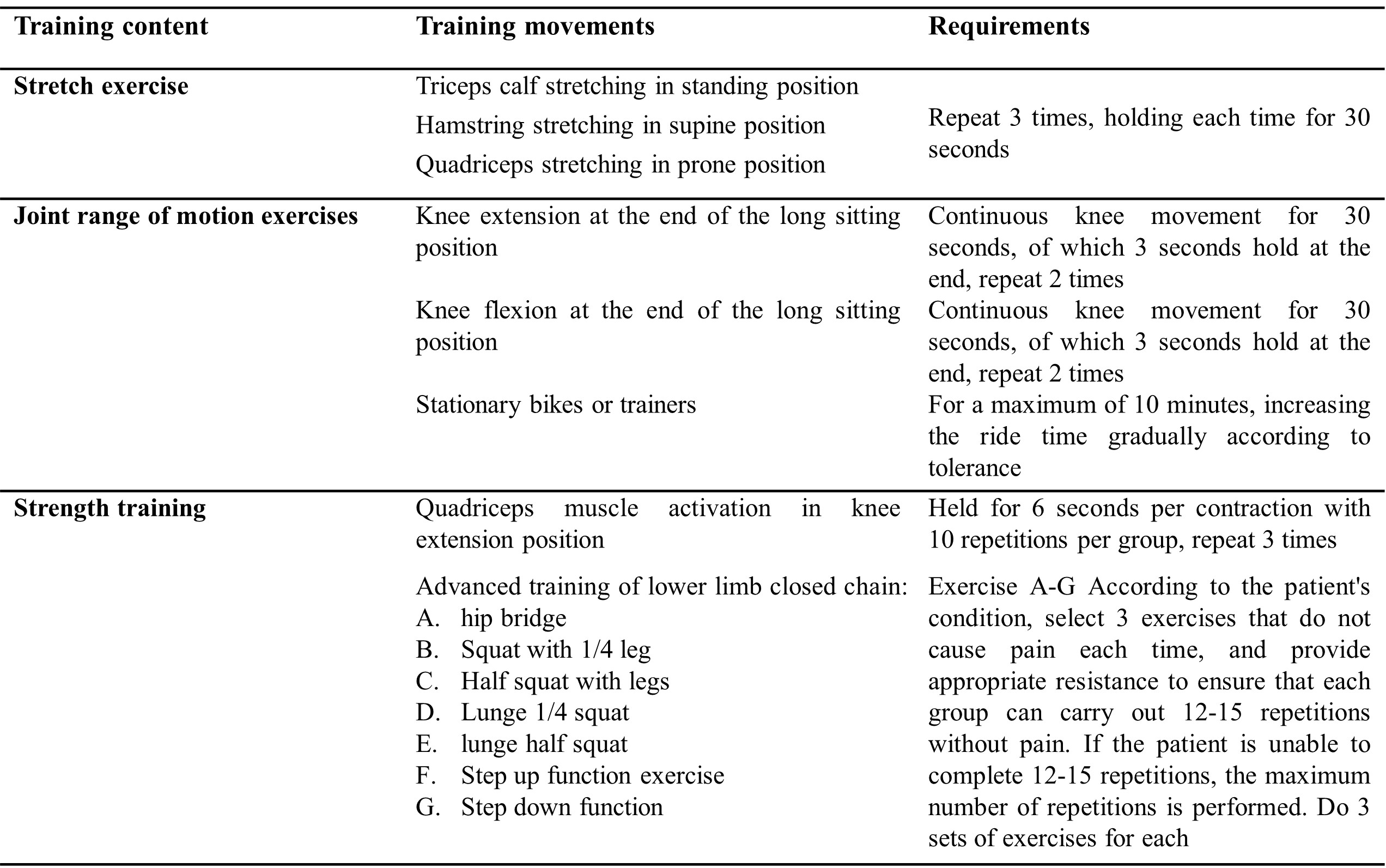

Supplement: Supplementary Figure 1 — Rehabilitation program. [file Image1.jpeg]
