# Supplementary material for: Different intensities of aerobic training for patients with type 2 diabetes mellitus and knee osteoarthritis: a randomized controlled trial
Source: Front Endocrinol (Lausanne). 2024 Sep 2;15:1463587. doi: 10.3389/fendo.2024.1463587 (PMC11402742; doi:10.3389/fendo.2024.1463587)
Supplement: Supplementary Table 1 — Primary and key secondary outcomes at 6 months in the per-protocol population. [file Table1.doc]

S1. Table. Primary and key secondary outcomes at 6 months in the per-protocol population

| **Outcome** | **High-intensity group (N=71)** | **Moderate-intensity group (N=72)** | **Control group (N=69)** | **Mean difference (95% CI)** | **P value** |
| --- | --- | --- | --- | --- | --- |
| **Primary outcomes** |  |  |  |  |  |
| **HbA1c level** |  |  |  |  |  |
| 6-mo adjusted means from baseline (95% CI)* | 1.12 (0.92 to 1.32) | 0.76 (0.58 to 0.94) | 0.66 (0.49 to 0.84) |  |  |
| High-intensity vs control |  |  |  | 0.46 (0.12 to 1.05) | .02 |
| High-intensity vs moderate-intensity |  |  |  | 0.36 (0.05 to 0.82) | .04 |
| Moderate-intensity vs control |  |  |  | 0.10 (-0.20 to 0.42) | .06 |
| **Key secondary outcomes** |  |  |  |  |  |
| **KOOS pain** |  |  |  |  |  |
| 6-mo adjusted means from baseline (95% CI) | 3.4 (2.9 to 3.9) | 2.9 (2.0 to 3.8) | 1.7 (1.2 to 2.2) |  |  |
| High-intensity vs control |  |  |  | 1.6 (0.4 to 2.8) | .02 |
| High-intensity vs moderate-intensity |  |  |  | 0.5 (-0.7 to 1.7) | .31 |
| Moderate-intensity vs control |  |  |  | 1.2 (0.1 to 2.3) | .04 |
| **KOOS QoL** |  |  |  |  |  |
| 6-mo adjusted means from baseline (95% CI) | 5.8 (3.9 to 7.7) | 4.7 (3.4 to 5.0) | 2.4 (1.6 to 3.2) |  |  |
| High-intensity vs control |  |  |  | 3.4 (0.9 to 5.9) | .02 |
| High-intensity vs moderate-intensity |  |  |  | 1.1 (-0.8 to 3.0) | .47 |
| Moderate-intensity vs control |  |  |  | 2.3 (0.1 to 4.5) | .04 |

Abbreviations: KOOS, Knee injury and Osteoarthritis Outcome Score; Qol, quality of life.

The model-adjusted outcomes used only participants with complete follow-up data.
